# The roles of α_V_ integrins in lens EMT and posterior capsular opacification

**DOI:** 10.1111/jcmm.12213

**Published:** 2014-02-04

**Authors:** Fahmy A Mamuya, Yan Wang, Victoria H Roop, David A Scheiblin, Jocelyn C Zajac, Melinda K Duncan

**Affiliations:** Department of Biological Sciences, University of DelawareNewark, DE, USA

**Keywords:** lens, epithelial-to-mesenchymal transition, posterior capsular opacification, integrins, transforming growth factor beta, wound healing response, fibrosis, secondary cataract

## Abstract

Posterior capsular opacification (PCO) is the major complication arising after cataract treatment. PCO occurs when the lens epithelial cells remaining following surgery (LCs) undergo a wound healing response producing a mixture of α-smooth muscle actin (α-SMA)-expressing myofibroblasts and lens fibre cells, which impair vision. Prior investigations have proposed that integrins play a central role in PCO and we found that, in a mouse fibre cell removal model of cataract surgery, expression of α_V_ integrin and its interacting β-subunits β1, β5, β6, β8 are up-regulated concomitant with α-SMA in LCs following surgery. To test the hypothesis that α_V_ integrins are functionally important in PCO pathogenesis, we created mice lacking the α_V_ integrin subunit in all lens cells. Adult lenses lacking α_V_ integrins are transparent and show no apparent morphological abnormalities when compared with control lenses. However, following surgical fibre cell removal, the LCs in control eyes increased cell proliferation, and up-regulated the expression of α-SMA, β1-integrin, fibronectin, tenascin-C and transforming growth factor beta (TGF-β)–induced protein within 48 hrs, while LCs lacking α_V_ integrins exhibited much less cell proliferation and little to no up-regulation of any of the fibrotic markers tested. This effect appears to result from the known roles of α_V_ integrins in latent TGF-β activation as α_V_ integrin null lenses do not exhibit detectable SMAD-3 phosphorylation after surgery, while this occurs robustly in control lenses, consistent with the known roles for TGF-β in fibrotic PCO. These data suggest that therapeutics antagonizing α_V_ integrin function could be used to prevent fibrotic PCO following cataract surgery.

## Introduction

Cataract is a clouding of the ocular lens, which is the most common cause of blindness in the world 1 with 43% of the 44.8 million blind suffering from cataract 2. Cataracts are treated by an outpatient surgical procedure that involves extracapsular cataract extraction (ECCE) followed by implantation of an artificial intraocular lens (IOL) 3,4. This procedure is the most commonly performed outpatient surgery performed in the USA and has successfully restored the vision of over 30 million cataract patients in the USA alone. It is projected that 30 million cataract surgeries per year will be done globally by the year 2020 5. However, this surgical volume means that even low complication rates have a significant societal impact, because of both increased medical costs and visual disability.

The most common negative outcome of cataract surgery is posterior capsular opacification (PCO), which is reported to occur following 3–100% of cataract surgeries depending on numerous factors including patient age, surgical method, type of IOL implanted and post-surgical follow-up time 6. As most IOL designs are implanted within the lens basement membrane (lens capsule) to fix the IOL in the optical path, most of the lens capsule is left intact in the eye. However, it is not possible to completely remove the lens epithelial cells (LECs) attached to the intact lens capsule, and these residual lens cells (LCs) initiate a wound healing response that includes cell proliferation, migration following surgery. Some of these cells also undergo an epithelial–mesenchymal transition (EMT) into migratory myofibroblasts, while others undergo lens fibre cell differentiation in an attempt to regenerate the lens 7,8. If these LCs, which at this point of time are no longer phenotypically normal LECs, remain outside of the visual axis, a Soemmering's ring results, which can stabilize some of the current IOL designs within the eye 9. However, if they migrate along the lens capsule into the visual axis, PCO results, leading to impairment of vision 10. Substantial efforts have been made to prevent and treat PCO, leading to a reduction in its incidence shortly following cataract surgery 11. However, longer term, PCO, is still a major barrier to the long-term restoration of high acuity vision in cataract patients 6.

Numerous studies suggest that transforming growth factor beta (TGF-β) signalling is crucial for the pathogenesis of fibrotic PCO deriving from the EMT of LCs 7,12. However, less is known about how cataract surgery initiates TGF-β–mediated EMT as high levels of latent TGF-β are constitutively present in the ocular fluids 13.

Integrins are heterodimeric extracellular matrix (ECM) receptors consisting of one α- and one β-integrin subunit, which play major roles in cell proliferation, adhesion and migration of numerous cell types 14,15. Integrins are expressed by both lens epithelial and fibre cells and play major roles in lens development and epithelial cell survival 16,17. Furthermore, some integrins have been reported to up-regulate in LCs after injury or surgery and integrin antagonists can attenuate this process 18–20. However, the identity of the particular integrins involved and their function still remains uncertain.

In this study, we demonstrate that integrins belonging to the α_V_ family are not essential for lens maturation or homoeostasis, but are up-regulated at the protein level in the LCs remaining on the lens capsule in a mouse model of cataract surgery. Furthermore, LCs lacking the α_V_ integrin gene fail/delay entrance into the EMT that causes fibrotic PCO. As therapeutics targeting α_V_ integrins have been developed and are in clinical trials for other diseases 21, these findings suggest that such drugs are potential therapeutics for PCO prevention.

## Materials and methods

### Mice

All the experiments conducted conform to the ARVO Statement for the Use of Animals in Ophthalmic and Vision Research and were approved by the University of Delaware Institutional Animal Care and Use Committee. All mice were bred and maintained under pathogen-free conditions at the University of Delaware animal facility under a 14/10-hr light/dark cycle. C57BL/6 mice carrying an α_V_ integrin allele with loxP sites flanking exon 4 (α_V_^[+/flox]^) 22 were obtained from Adam Lacy-Hulbert (Harvard School of Medicine, Boston, MA). FVB/N mice expressing *cre*-recombinase in all LCs from the lens vesicle stage onward (MLR10-*cre*) 23 were obtained from Michael L. Robinson (Miami University, Oxford, OH, USA) and backcrossed 10 generations to C57BL/6<har> mice to create a congenic line. Homozygous male FVB/N mice carrying a *cre*-recombinase gene under the control of the adenovirus EIIA-promoter, *EIIa-cre/EIIa-cre* (TgN(EIIa-Cre)C5379Lmgd) 24, were obtained from The Jackson Laboratories Bar Harbor, Maine. Heterozygous α_V_ integrin flox mice (α_V_^[flox/+]^) were mated to the *EIIa-cre/EIIa-cre* mice to generate mice carrying a germline α_V_ integrin null allele α_V_^[−/+]^. These animals were mated to α_V_^[flox/flox]^ to generate α_V_^[−/flox]^ mice. α_V_^[−/flox]^ were mated to MLR10-*cre* mice to generate mice lacking α_V_ integrin in their entire lens α_V_^[−/flox]^; MLR10-*cre* (αVMLR10). All control mice used in this study are αv ^[flox/flox]^ that lack a *cre*-recombinase expressing transgene. All embryos were staged based on E0.5 being the day that a vaginal plug was found in the dam.

### PCR and genotyping

DNA was isolated from half centimetre cut tail snips or one whole lens using the PureGene Tissue and Mouse Tail kit (Gentra Systems, Minneapolis, MN, USA). The animals were genotyped by PCR using primers described in Table 1.

**Table 1:** List of all primers used for both PCR and qRT-PCR

	Gene/cDNA detected	Forward primer	Reverse primer
1	α_V_ integrin [null allele]	5′-GGTGACTCAATCGACCTTCAGC	5′-CAGAAATCAAGGACCAAACTGAG
2	α_V_ integrin [floxed]	5′-TTCAGGACGGCACAAAGACCGTTG	5′-CACAAATCAAGGATGACCCTGAG
3	α_V_ integrin [qrt-pcr]	5′-GATGCAGTGTGAGGAACTGGT	5′-GAGTGAACTGGTTCAGGATGG
4	α-SMA	5′-GCACAGCTTCTCCTTGATGTC	5′-5′CCGAGATCTCACCGACCT
5	β2M	5′-TACGCCTGCAGAGTTAAGCAT	5′-TCAAATGAATCTGAGCATCA
6	β1 integrin	5′-TCCTTCAATTGCTCACCTTGT	5′-GCGCACTGCTGACTTAGGAAT
7	β5 integrin	5′-AGGATCTACGGACCTTTCTGC	5′-CATTTGCATTCTCCACAGTGA
8	β6 integrin	5′-GCAGAACGCTCTAAGGCCAA	5′- AAAGTGCTGGTGGAACCTCG
9	β8 integrin	5′-AAGCAAAGGCTGTCCAGTTG	5′-TCCACGGGGTATTTCTTCAG
10	Cre-recombinase	5′-ATGCTTCTGTCCGITTGCCG	5′-CTTGTTTTGCACGTTCACCG
11	Fibronectin	5′-CTGGAGTCAAGCCAGACACA	5′-CGAGGTGACAGAGACCACAA
12	Tenascin -C	5′-AAAGTAACCACAACCCGCCT	5′-AGGTGATCAGTGCTGTGGTG
13	TGF-β–induced protein	5′-CCTCACCTCCATGTACCAGAA	5′-TGGAAATGACCTTGTCAATGAG
14	Vitronectin	5′-CAAAGCTCGCACTGACA	5′-CCCCTGAGGCCCTTTTTCATA

### Gross morphological and optical analysis

Eyes were removed from 6-month-old mice and lenses were dissected. Lens transparency was assessed by placing lenses in Corning Cellgro Medium 199 (Mediatech Inc, Manassas VA) at 37°C and photographs taken under both bright-field and dark-field conditions using a Cannon digital camera A420 mounted on a Zeiss Stemi SV 11 Apo Stereo Microscope (Zeiss, Thornwood, NY, USA). For optical analysis, lenses were placed on a 200-mesh electron microscopy grid and photographed as previously described 25. The weight and dry mass of lenses was determined by weighing freshly isolated lenses, placing them in a 50°C oven for 96 hrs and then determining their dry weight. All assays were performed on at least three biological replicates.

### Scanning electron microscopy

Eyes were immersion fixed in 0.08 M Sodium Cacodylate buffer pH 7.4 (Electron Microscopy, Hatfield, PA, USA), 1.25% glutaraldehyde (Electron Microscopy) and 1% paraformaldehyde for 5 hrs (Electron Microscopy). The lens was excised and transferred to fresh fixative for an additional 48 hrs. After fixation, lenses were washed in 1× PBS. The lens capsule was peeled and the superficial fibre cell layers were removed with fine forceps to expose the cortical fibre cells; some lenses were peeled further to expose the nuclear fibre cells. Peeled lenses were subjected to an ethanol dehydration series (25%, 50%, 75% and 100%) followed by overnight incubations in fresh 100% ethanol followed by an additional two 2.5-hr 100% ethanol incubation. Finally, the dehydrated lenses were critical point dried using hexamethyldisilazane (Electron Microscopy) as previously described 26, mounted on aluminium stubs and coated with gold/palladium for 2.5 min. Samples were viewed with a field emission scanning electron microscope Hitachi S-4700 (Tokyo, Japan) 27.

### Surgical removal of lens fibre cells

The effect of cataract surgery on LCs was modelled in living mice by surgical removal of lens fibre cells as previously described 28,29. Briefly, 3-month-old mice were anesthetized, a central corneal incision was made and the entire lens fibre cell mass was removed by a sharp forceps, leaving behind an intact lens capsule. The corneal incision was closed with a single 10-0 nylon corneal suture and normal saline was injected to inflate the eye back to its normal shape. For analysis, mice were killed at various time intervals after surgery ranging from 24 hrs to 5 days. Time zero controls were obtained by re-anesthetizing previously operated mice and the extracapsular lens extraction procedure was performed in the contralateral eye from the first surgery just prior to killing. This minimized the number of animals used for these experiments as no changes in marker expression were observed between time zero samples obtained from naïve mice and those whose other eye had previously undergone lens fibre cell removal. At least five independent animals were used for each analysis.

### Immunofluorescence

All immunofluorescence analyses were carried out as previously described 30. Briefly, to confirm α_V_ integrin deletion, heads were removed from embryos and eyes removed from post-natal mice and embedded directly in optimum cutting temperature (OCT) media (Tissue Tek, Torrance, CA, USA). For analysing post-surgery samples, operated eyes were collected at 0 hr, 48 hrs and 5 days after surgery, embedded in OCT and directly stored at −80°C. Sixteen-micron-thick sections were obtained with a Leica CM3050 cryostat (Leica Microsystems, Buffalo Grove, IL, USA) and mounted on glass slides followed by a 1:1 acetone:methanol fixation at −20°C for 20 min. or in 4% paraformaldehyde fixation for 30 min. at room temperature. Depending on the antibody, appropriate blocking sera were diluted in either 1× PBS or 1× Tris-buffered saline (TBS), and slides were blocked for 1 hr at room temperature. Primary antibodies diluted in blocking serum (see Table 2 for antibodies and dilutions used) were applied onto the slide and incubated in a humid chamber at room temperature for another 1 hr or overnight at 4°C. Slides were washed in either 1× PBS or 1× TBS at room temperature and incubated with a 1:2000 dilution of Draq5 (Biostatus, Leicestershire, UK), fluorescein labelled anti-αSMA (Sigma-Aldrich, St. Louis, MO, USA) along with the appropriate AlexaFluor 568 labelled secondary antibodies (Invitrogen, Carlsbad, CA, USA) for 1 hr at room temperature.

**Table 2:** List of antibodies used in this study

Primary antibody name	1-hr room temperature blocking conditions	Primary antibody incubation	Company and location
α-SMA Clone 1A4, F3777	1% BSA in PBS	1 hr at RT 1:250 dilution	Sigma-Aldrich
αV integrin AB1930	5% Horse serum + 5% Goat serum in PBS	Overnight at 4°C 1:400 dilution	Millipore (Billerica, MA, USA)
β1 integrin Clone MB1.2, 1997	1% BSA in PBS	1 hr at RT 1:250 dilution	Millipore
β5 integrin AB 1925	1% BSA in PBS	1 hr at RT 1:200 dilution	Chemicon (Temecula, CA, USA)
β6 integrin (H-1100): sc-15329	5% Goat serum in PBS	Overnight at 4°C 1:100 dilution	Santa Cruz (Santa Cruz, CA, USA)
β8 integrin Pro Sci Xw 7802	1% BSA in PBS	1 hr at RT 1:200 dilution	Chemicon
Phospho-SMAD 3 Clone EP823Y	10 min. wash in 5% BSA followed by 5% Goat serum + 10% Horse serum in PBS	Overnight at 4°C 1:100 dilution	Epitomics (Burlingame, CA, USA)
TGF-β-induced (H58) sc-28660	5% Goat serum in PBS	1 hr at RT 1:100 dilution	Santa Cruz
Fibronectin Ab23750	1% BSA in PBS	1 hr at RT 1:400 dilution	Abcam (Cambridge, MA, USA)
Vitronectin Ab28023	10% Goat serum in TBS	1 hr at RT 1:500 dilution	Abcam
Tenascin-C AB19011	5% Goat serum in PBS	Overnight at 4°C 1:400 dilution	Millipore
Prox 1 26	1% BSA in PBS	1 hr at RT 1:500 dilution	University of Delaware (Newark, DE, USA)
cMaf sc7866	1% BSA in PBS	1 hr at RT 1:100 dilution	Santa Cruz
Caspase -3 (Asp175)# 8120	5% Goat serum in TBS	Overnight at 4°C 1:50 dilution	Cell Signaling (Danvers, MA, USA)

### Confocal microscopy

Slides were washed, cover slipped and imaged with a Zeiss LSM 780 Confocal Microscope (Carl Zeiss, Inc., Gottingen, Germany) equipped with a 405-nm diode laser, an Argon laser with 458/488/514 nm lines, DPSS 561 nm and HeNe 633 nm laser. All comparisons of staining intensity between specimens were performed on sections stained simultaneously and the imaging for each antibody was performed with identical laser power and software settings to ensure validity of intensity comparisons. In some cases, images were processed after imaging to optimize brightness and contrast for viewing on diverse computer screens. In all cases, such manipulations were applied identically to experimental and control images.

### Proliferation assays

The number of LECs, which are in S phase at different times after surgery, were determined using 5-ethynyl-2′-deoxyuridine (EdU) click-it proliferation assays (Invitrogen, Grand Island, NY, USA). Briefly, mice were injected intraperitoneally with 400 μg/g bw of EdU dissolved in normal saline. Two hours later, the animals were killed; ocular tissue was embedded in OCT; and 16-μm frozen sections were obtained by cryostat and mounted on glass slides. If needed, slides were stored at −80°C or right away fixed by 4% paraformaldehyde for 30 min. followed by methanol fixation at −20°C for 10 min. Sections were allowed to dry and the EdU click-it reaction was carried out as previously described to detect EdU incorporated into DNA 31,32. The fluorescent signal was detected on a confocal microscope as described above.

### qRT-PCR

RNA was extracted from capsular bags obtained from 3-month-old mice collected at 0, 24 and 48 hrs after surgery using the SV Total RNA Isolation System (Promega, Madison, WI, USA). cDNA synthesis was carried out using the SA Biosciences RT^2^ First Strand Kit (Qiagen Inc., Valencia, CA, USA). Real-time PCR was performed with a QuantiTect SYBR Green PCR Kit (Qiagen Inc.) using an ABI7300 Real-Time PCR system (Applied Biosystems, Foster City, CA, USA) using a standard cycle temperature set of 50°C for 2 min., 95°C for 10 min., 95°C at 15 sec. for 45 cycles followed by 60°C for 1 min. mRNA levels for each gene were normalized to the expression levels of the housekeeping gene β2-microglobulin (β2M). Table 1 shows a list of all primers used.

### Micro RNA assay

For small RNA analysis, total RNA was isolated from capsular bags collected at 0 and 24 hrs after surgery from 3-month-old mice. Reverse transcription was performed by Eppendorf MasterCycler using a TaqMan® microRNA Reverse Transcription kit (Taqman® Small RNA Assays from Life Technologies, Carlsbad, CA, USA) to make cDNA. The levels of mir31 were compared using SnoRNA202 as the housekeeping control (Taqman® Small RNA Assays from Life Technologies, Carlsbad, CA, USA). Quantitative real-time PCR was performed with an ABI Prism 7300 Sequence Detection System under TaqMan® Small RNA Assay protocol guide.

### Statistics

All statistics were assessed using either Student's *t*-test or one-way anova with Tukey's post hoc test. Differences were considered significant at *P *<* *0.05.

## Results

### α_V_ integrin and its interacting β-subunits are overexpressed 48 hrs after surgery in lens

It has been previously postulated that α_V_ integrins play a major role in lens epithelial cell EMT 18,17, although little experimental evidence for this idea has been reported. To characterize the distribution of α_V_ integrins in residual LECs after a lens injury similar to cataract removal, we used a mouse surgical model of ECCE that was first reported in 29 and refined as described in 28.

**Figure 1:**
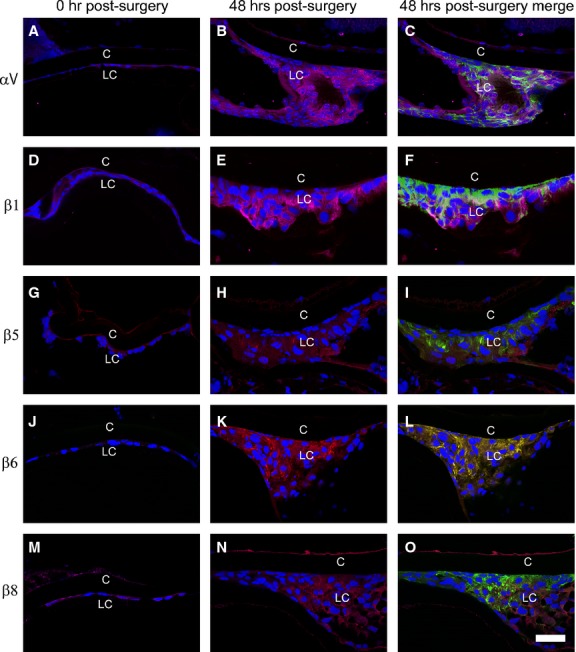
Immunofluorescent analysis showing that α-smooth muscle actin (α-SMA) and α_V_-β integrin levels increase in wild-type residual lens cells (LC) at 48 hrs after surgery (A) α_V_ integrin + α-SMA expression, 0 hr after surgery. (B) α_V_ integrin expression at 48 hrs after surgery. (C) α_V_ integrin + α-SMA expression, 48 hrs after surgery. (D) β1 integrin + α-SMA expression, 0 hr after surgery. (E) β1 integrin expression, 48 hrs after surgery. (F) β1 integrin + α-SMA expression, 48 hrs after surgery. (G) β5 integrin + α-SMA 0 hr after surgery. (H) β5 integrin expression at 48 hrs after surgery. (I) β5 integrin + α-SMA expression at 48 hrs after surgery. (J) β6 integrin + α-SMA expression 0 hr after surgery. (K) β6 integrin expression at 48 hrs after surgery. (L) β6 integrin + α-SMA expression 48 hrs after surgery. (M) β8 integrin + α-SMA expression 0 hr after surgery. (N) β8 integrin expression at 48 hrs after surgery. (O) β8 integrin + α-SMA expression, 48 hrs after surgery. Scale bar = 35 μm, red = integrin; blue = nucleus; green = α-SMA, LC = residual lens cells, C = lens capsule.

**Figure 2:**
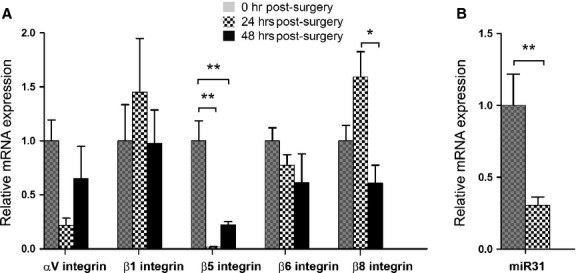
(A) RT-PCR quantitation of integrin mRNA levels in wild-type residual lens cells (LC) at 0, 24 and 48 hrs after surgery normalized to β2-microglobulin *N* = 4. αV integrin subunit mRNA expression appeared attenuated at 24 hrs, but this did not reach significance (*P* = 0.083). β5 integrin subunit mRNA expression was significantly reduced at both 24 and 48 hrs after surgery (**P < 0.0014). No significant changes were observed in β1 or β6 integrin subunit mRNA expression after surgery. β8 integrin subunit mRNA appeared to increase slightly at 24 hrs, although the difference was not significant. Its abundance did significantly fall at 48 hrs after surgery (*P < 0.014). (B) Quantitation of miR-31 mRNA levels in wild-type LCs at 24 hrs after surgery normalized to snoRNA202 levels (**P < 0.0087, *N* = 5). All fold changes after surgery were calculated by setting values obtained at 0 hr after surgery in each specific group to one. Values are expressed as mean ± SEM. Asterisks (*) indicate statistically significant fold changes from 0 hr after surgery.

By immunofluorescence, we found that the α_V_ integrin subunit is only modestly expressed in LCs at the time of surgery, but its levels up-regulated dramatically by 48 hrs after surgery in cells that exhibit the multilayering and increased α-SMA expression associated with PCO (Fig. 1A–C). In addition to the α_V_ integrin subunit, four of α_V_ integrin's interacting β subunits, β1, β5, β6 and β8 integrin subunits, were also overexpressed by 48 hrs after surgery (Fig. 1D–O). In contrast, the levels of α5 and α6 integrin were only slightly up-regulated, while α1, α2, α3, β2 and β3 integrin protein levels did not change or were undetectable over this time frame (data not shown). Notably, the up-regulation of α_V_ integrin expression was only observed at the protein level, as the mRNA levels for αV integrin and its interacting β-integrins either did not change significantly or decreased significantly by 48 hrs after surgery (Fig. 2A). This effect could be mediated by microRNAs as miR31, a microRNA known to regulate integrin translation 32,33, did down-regulate significantly by 24 hrs after surgery (Fig. 2B).

### Deletion of α_V_ integrin from the developing lens

**Figure 3:**
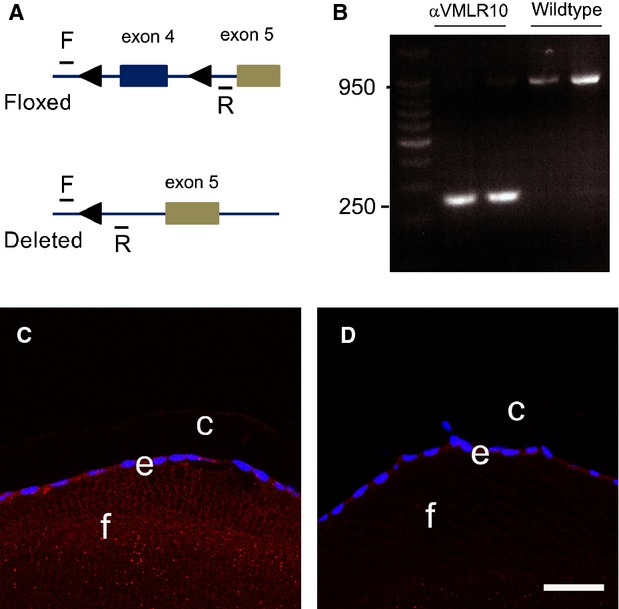
α_V_ integrin gene deletion analysis. (A) Diagram of the α_V_ integrin locus showing the position of the PCR primers and the loxP sites 22. (B) PCR results from DNA obtained from 3-month-old lenses demonstrating successful deletion of exon 4 in mice lacking α_V_ integrin in all lens cells α_V_^[−/flox]^; MLR10-*cre* (αVMLR10). (C) Immunofluorescence showing α_V_ integrin protein expression in a 3-month-old wild-type lens. (D) Immunofluorescence showing α_V_ integrin protein expression in a 3-month-old α_V_MLR10 lens Key: Scale = 35 μm. Red = α_V_ integrin, blue = nucleus, e = epithelial lens cells, f = lens fibre cells and c = lens capsule.

To study the role of α_V_ integrin in the lens during its development and in PCO, we created mice lacking α_V_ integrin solely in the lens using MLR10-*cre* whose activity is first detected in the lens beginning around embryonic day 10.5 (the lens vesicle stage) 23. PCR analysis of genomic DNA isolated from adult lenses showed that the deletion of the floxed region of the α_V_ integrin gene is nearly complete (Fig. 3A and B) and immunofluorescence analysis revealed significant reduction in α_V_ integrin protein beginning at about 12.5 dpc (data not shown) with the near total loss of the protein in adult α_V_^[−/flox]^; MLR10-*cre* (αVMLR10) lenses when compared with α_V_^[flox/flox]^ (wild-type; Fig. 3C and D).

### α_V_ integrin null lenses are morphologically and optically indistinguishable from wild-type

**Figure 4:**
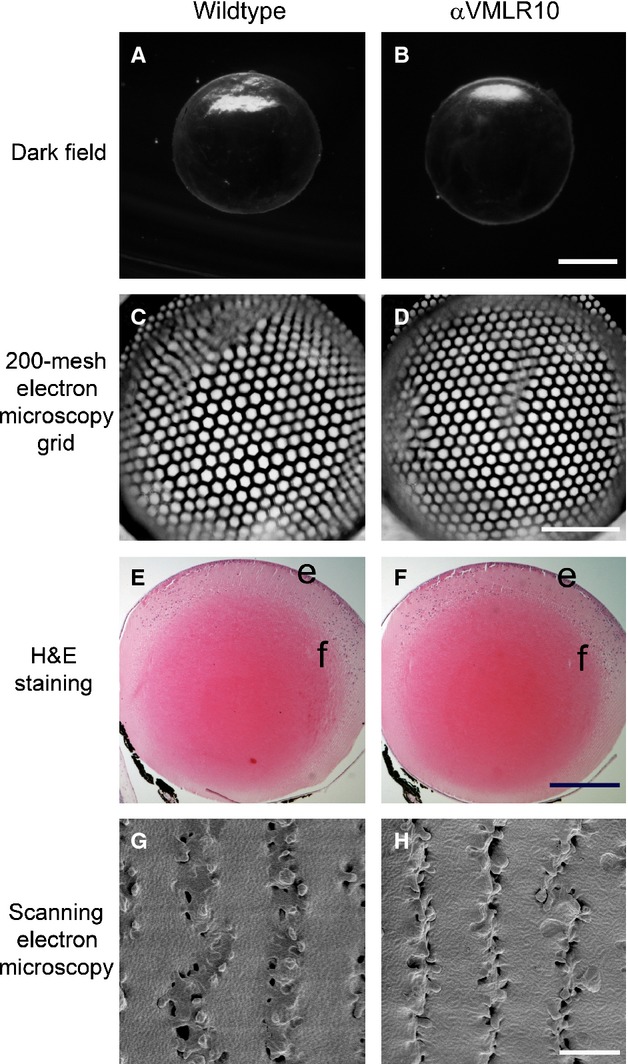
Morphological analysis of α_V_ integrin null lenses. (A) A dark-field image showing a 3-month-old wild-type lens. (B) A dark-field image showing a 3-month-old αVMLR10 lens. (C) A 200-mesh electron microscopy grid analysis of a 4-month-old wild-type lens. (D) A 200-mesh electron microscopy grid analysis of a 4-month-old αVMLR10 lens. (E) Haematoxylin and eosin staining showing 4-month-old wild-type lens. (F) Haematoxylin and eosin staining showing 4-month-old αVMLR10 lens. (G) SEM analysis of the fibre cell organization of a 4-month-old wild-type lens. (H) SEM analysis of the fibre cell organization of a 4-month-old αVMLR10 lens fibre cell organization. Scale bar for (A, B) = 1.0 mm, (C, D) = 0.5 mm, (E, F) = 0.5 mm and (G, H) = 4.0 μm; e = lens epithelium, f = lens fibre cells.

Lenses lacking α_V_ integrin appear transparent under dark-field imaging (Fig. 4A and B) and refracted a hexagonal grid similarly (Fig. 4B and C) suggesting that α_V_ integrin is not important for the transparency or refractive properties of the lens. At the light level, both wild-type and αVMLR10 lenses exhibit similar morphology (Fig. 4E and F) and no obvious defects in lens fibre cell structure were observed by scanning electron microscopy (Fig. 4G and H). αVMLR10 null lenses weighed significantly more than controls at 3 months of age, although by 6 months, this difference was no longer statistically significant (Table 3). However, the basis for this observation remains unclear as the ratio of wet lens to dry lens weight between αVMLR10 and wild-type is unchanged (Table 3) and no differences in cell proliferation were detected (data not shown).

**Table 3:** Lens wet and dry weight comparison between 4-month-old α_V_^[−/flox]^; MLR10-*cre* (αVMLR10) lenses and wild-type lenses

Lens age and genotype	Average wet lens weight (mg)	Average dry lens weight (mg)	Wet lens/dry lens ratio
3-month-old αVMLR10 lenses	7.78 ± 0.35^*^	3.54 ± 0.24	2.26
3-month-old wild-type lenses	7.26 ± 0.42^*^	3.29 ± 0.18	2.21
6-month-old αVMLR10 lenses	8.67 ± 0.30	4.12 ± 0.19	2.10
6-month-old wild-type lenses	8.44 ± 0.49	3.91 ± 0.20	2.16

αVMLR10 lenses are significantly heavier than wild-type at 3 months of age (^*^P = 0.02; *n* = 8). However, by 6 months of age, these lenses were no longer significantly different in size (*P* = 0.30, *n* = 6). In addition, the ratio of wet lens to dry lens between wild-type and αVMLR10 lenses was similar. All results are expressed on a per lens basis.

### LCs from lenses lacking αV integrin do not elevate cell proliferation and α-SMA expression 48 hrs after surgery

**Figure 5:**
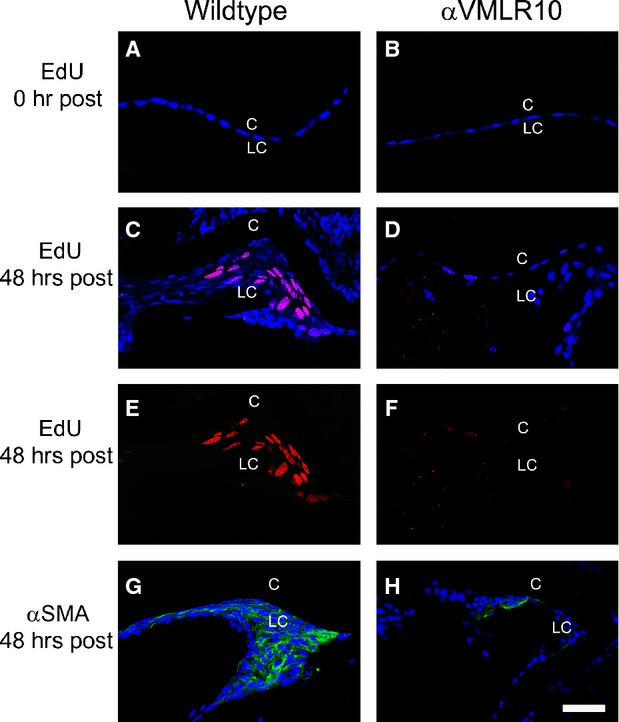
Immunohistochemistry analysis of α-smooth muscle actin (α-SMA) expression and 5-ethynyl-2′-deoxyuridine (EdU) click-it labelling of wild-type and αVMLR10 residual lens cells (LC) after surgery. (A) EdU labelling + α-SMA expression in wild-type LCs at 0 hr after surgery. (B) EdU + α-SMA expression in αVMLR10 LCs at 0 hr after surgery. (C) EdU staining of proliferating wild-type LCs at 48 hrs after surgery. (D) EdU labelling of αVMLR10 LCs at 48 hrs after surgery. (E) EdU labelling alone in proliferating wild-type LCs at 48 hrs after surgery. (F) EdU labelling alone in αVMLR10 LCs at 48 hrs after surgery. (G) α-SMA expression in wild-type LCs at 48 hrs after surgery. (H) α-SMA expression on αVMLR10 LCs at 48 hrs after surgery. Scale bar (A–F) = 70 μm, (G, H) = 35 μm. Red = EdU positive (Proliferating LCs), blue = nucleus, green = α-SMA, LC = residual lens cells, C = lens capsule.

As the absence of α_V_ integrin from lens did not obviously affect normal lens development, morphology or function, we then used αVMLR10 mice as a model to study the role of α_V_ integrin in the cellular and molecular changes that occur in LCs after lens injury/during PCO. Immediately after fibre cell removal, the LCs in both wild-type (Fig. 5A) and αVMLR10 mice (Fig. 5B) exhibit neither appreciable cell proliferation nor do they express the EMT marker, α-SMA. However, 48 hrs later, the LCs of wild-type lenses proliferate (Fig. 5C and E) and initiate robust α-SMA expression (Fig. 5G). In contrast, the residual LCs remaining in αVMLR10 capsule after fibre cell removal exhibit very little cell proliferation (Fig. 5D and F) and do not appreciably up-regulate α-SMA expression by 48 hrs after surgery (Fig. 5H). Apoptosis, as assayed by active caspase-3 levels, was not detected in either wild-type or αVMLR10 lenses after lens fibre cell removal (data not shown).

### αVMLR10 lenses do not up-regulate EMT markers in LCs remaining in the eye after fibre cell removal

**Figure 6:**
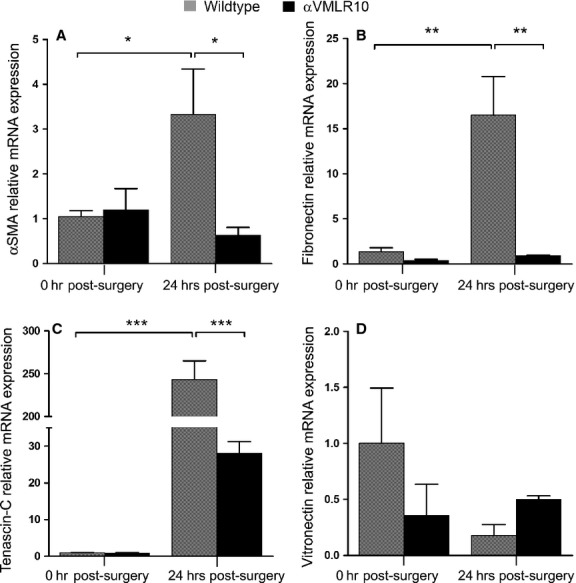
RT-PCR quantification of mRNA expression levels in wild-type and αVMLR10 residual lens cells on capsular bags collected at 0 and 24 hrs after surgery. For each gene, mRNA expression was normalized to β2M and fold change was calculated based on the mean 0 hr after surgery, wild-type mRNA level equated to 1. (A) α-smooth muscle actin (α-SMA) relative mRNA expression after surgery, *P < 0.02. There was no significant changes in α-SMA mRNA levels between 0 and 24 hrs αVMLR10 post-surgery lenses, *P* = 0.54 (B) Fibronectin relative mRNA expression after surgery, **P < 0.001. There was no significant changes in fibronectin mRNA levels between 0 and 24 hrs αVMLR10 post-surgery lenses, *P* = 0.90 (C) Tenascin-C relative mRNA expression after surgery ***P < 0.0001. There was no significant increase in tenascin-C mRNA expression in αVMLR10 24 hrs post-surgery lenses, *P* = 0.08 (D) No significant changes were observed in vitronectin relative mRNA expression in any group after surgery when compared with wild-type 0 hr, *P* = 0.21. The decrease in vitronectin mRNA expression in wild-type at 24 hrs after surgery was not significant, *P* = 0.79; neither was the slight increase in vitronectin mRNA expression in αVMLR10 at 24 hrs after surgery, *P* = 0.41. All experiments had *N* = 5. Values are expressed as mean ± SEM. Asterisks (*) indicate statistically significant fold changes from 0 hr after surgery.

The expression levels of α-SMA (Fig. 6A), fibronectin (Fig. 6B) and tenascin-C (Fig. 6C) up-regulate significantly in wild-type LCs during lens EMT as previously reported in human PCO 35–37. Interestingly, we found that α-SMA mRNA levels did not increase in αVMLR10 lenses 24 hrs after surgery (Fig. 6A), whereas the extent of fibronectin (Fig. 6B) and tenascin-C (Fig. 6C) up-regulation was greatly attenuated in lenses lacking the α_V_ integrin gene. In contrast though, the mRNA levels of the α_V_ integrin ligand vitronectin did not change significantly after surgery. Compared with wild-type, vitronectin mRNA levels were initially lower in αVMLR10 lenses and increased slightly at 24 hrs after surgery; however, none of these changes were statistically significant (Fig. 6D).

**Figure 7:**
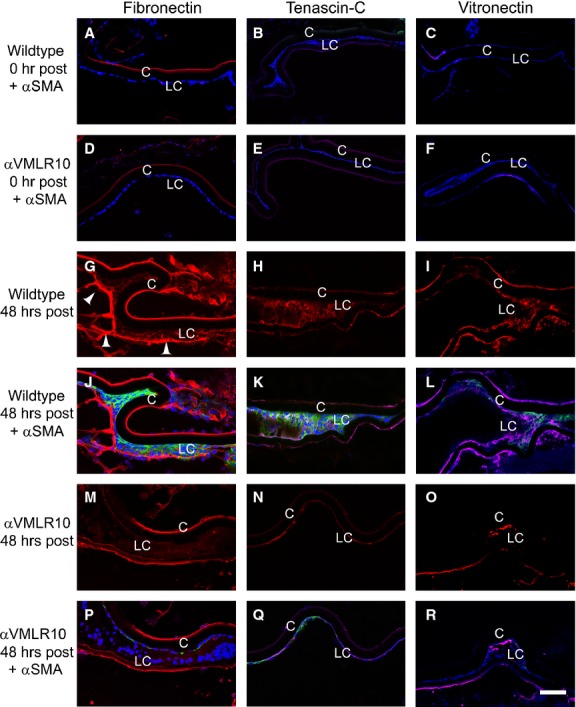
Immunofluorescent analysis of fibronectin, tenascin-C and vitronectin deposition in wild-type and αVMLR10 residual lens cells (LC) after surgery. (A) Fibronectin + α-smooth muscle actin (α-SMA) expression in wild-type LCs at 0 hr after surgery. (B) Tenascin-C + α-SMA expression in wild-type LCs at 0 hr after surgery. (C) Vitronectin + α-SMA expression in wild-type LCs at 0 hr after surgery. (D) Fibronectin + α-SMA expression in αVMLR10 LCs at 0 hr after surgery. (E) Tenascin-C + α-SMA expression in αVMLR10 LCs at 0 hr after surgery. (F) Vitronectin + α-SMA expression in αVMLR10 LCs at 0 hr after surgery. (G) Fibronectin expression in wild-type LCs 48 hrs after surgery with arrowheads showing fibronectin deposition on the leading LCs. (H) Tenascin-C expression alone in wild-type LCs at 48 hrs after surgery. (I) Vitronectin expression alone in wild-type LCs at 48 hrs after surgery. (J) Fibronectin + α-SMA expression in wild-type LCs at 48 hrs after surgery. (K) Tenascin-C + α-SMA expression in wild-type LCs at 48 hrs after surgery. (L) Vitronectin + α-SMA expression in wild-type LCs at 48 hrs after surgery. (M) Fibronectin expression in αVMLR10 LCs at 48 hrs after surgery. (N) Tenascin-C expression alone in αVMLR10 LCs at 48 hrs after surgery. (O) Vitronectin expression in αVMLR10 LCs at 48 hrs after surgery. (P) Fibronectin + α-SMA expression in αVMLR10 LCs at 48 hrs after surgery. (Q) Tenascin-C + α-SMA expression in αVMLR10 LCs at 48 hrs after surgery (R) vitronectin + α-SMA expression in αVMLR10 LCs at 48 hrs after surgery. Scale bar = 60 μm. Red = Fibronectin, tenascin-C or Vitronectin, blue = nucleus, green = α-SMA. LC = residual lens cells, C = lens capsule.

By immunofluorescence, fibronectin, tenascin-C and vitronectin proteins were nearly undetectable in both wild-type (Fig. 7A–C) and αVMLR10 (Fig. 7D–F) LCs immediately following and 24 hrs after surgery (data not shown here), but were elevated levels 48 hrs after surgery in wild-type (Fig. 7G–I) LCs, which are expressing α-SMA (Fig. 7J–L). However, αVMLR10 LCs show greatly attenuated fibronectin, tenascin-C and vitronectin (Fig. 7M–O) and relatively lower α-SMA elevations (Fig. 7P–R) 48 hrs after surgery. Immunofluorescence analysis also revealed that fibronectin, which is present in the lens capsule of both wild-type (Fig. 7A) and αVMLR10 (Fig. 7D) lenses prior to surgery, is not obviously elevated around LCs at 24 hrs after surgery (data not shown), but is deposited around LCs expressing α-SMA by 48 hrs after surgery especially on the leading LCs (Fig. 7G). However, such extensive fibronectin deposition was not observed in αVMLR10 LCs (Fig. 7M). Surprisingly, unlike the QRT-PCR results, vitronectin levels were up-regulated at 48 hrs after surgery in wild-type (Fig. 7I and L) but not in αVMLR10 lenses (Fig. 7O and R).

### Lens fibre differentiation markers still up-regulate after surgery in the absence of α_V_ integrin

**Figure 8:**
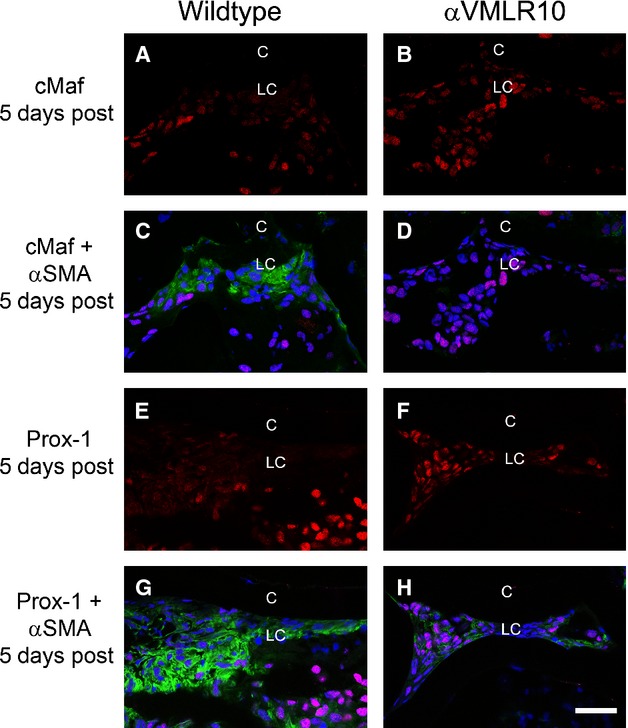
Immunohistochemistry of α-smooth muscle actin (α-SMA) and the lens fibre differentiation markers Prox-1 and cMaf in wild-type and αVMLR10 residual lens cells (LC) from capsular bags collected at 5 days after surgery. (A) cMaf expression alone in wild-type LCs at 5 days after surgery. (B) cMaf expression alone in αVMLR10 LCs at 5 days after surgery. (C) cMaf + α-SMA expression in wild-type LCs at 5 days after surgery. (D) cMaf + α-SMA expression in αVMLR10 LCs at 5 days after surgery. (E) Prox-1 expression alone in wild-type LC at 5 days after surgery. (F) Prox-1 expression alone in αVMLR10 LCs at 5 days after surgery. (G) Prox-1 + α-SMA expression in wild-type LCs at 5 days after surgery. (H) Prox-1 + α-SMA expression in αVMLR10 LCs at 5 days after surgery. Scale bar = 35 μm. Red = Prox-1 and cMaf, blue = nucleus, green = α-SMA, LC = residual lens cells, C = lens capsule.

After cataract surgery/lens injury, LCs do not exclusively undergo EMT. Instead, some LCs begin to express lens fibre cell markers presumably in an attempt to regenerate the injured lens 10,8,38. At 5 days after surgery in the mouse model used here, the residual wild-type LCs are found in cell clusters either expressing EMT markers such as α-SMA (Fig. 8C and G) or lens fibre cell markers such as cMaf (Fig. 8A and C) or Prox1 (Fig. 8E and G). However, similar to the results at 48 hrs after surgery, αVMLR10 LCs do not express appreciable α-SMA (Fig. 8D and H), although they do begin to express both cMaf (Fig. 8B and D) and Prox1 (Fig. 8F and H) similar to wild-type.

### αVMLR10 lenses fail to up-regulate SMAD-3 phosphorylation after surgery

**Figure 9:**
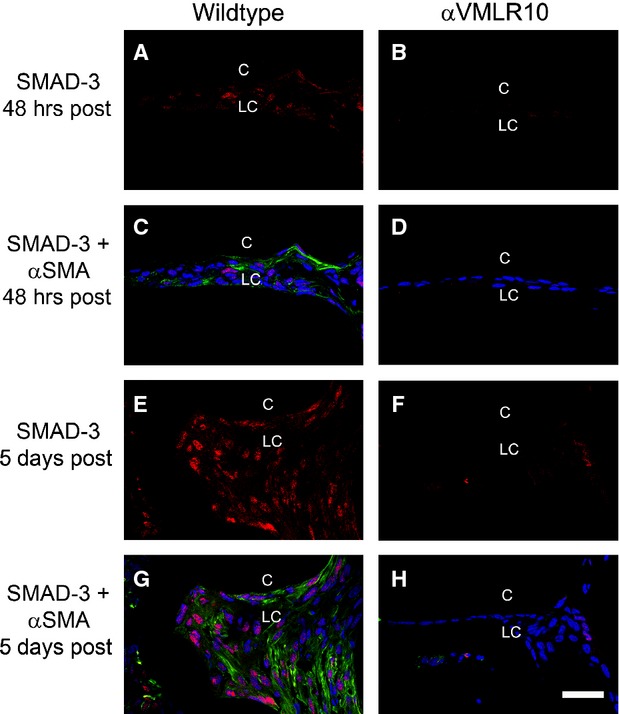
Immunofluorescent analysis of α-smooth muscle actin (α-SMA) and phospho-SMAD-3 in wild-type and αVMLR10 residual lens cells (LC) from capsular bags collected at 48 hrs and 5 days after surgery (A) Phospho-SMAD-3 expression alone in wild-type LCs at 48 hrs after surgery. (B) Phospho-SMAD-3 expression alone in αVMLR10 LCs at 48 hrs after surgery. (C) Phospho-SMAD-3 + α-SMA expression in wild-type LCs at 48 hrs after surgery. (D) Phospho-SMAD-3 + α-SMA expression in αVMLR10 LCs at 48 hrs after surgery. (E) Phospho-SMAD-3 expression alone in wild-type LCs at 5 days aftersurgery. (F) Phospho-SMAD-3 expression alone in αVMLR10 LECs at 5 days after surgery. (G) Phospho-SMAD-3 + α-SMA expression in wild-type LCs at 5 days after surgery. (H) Phospho-SMAD-3 + α-SMA expression in αVMLR10 LCs at 5 days after surgery. Scale bar = 35 μm. Red = phospho-SMAD-3, blue = nucleus, green = α-SMA, LC = residual lens cells, C = lens capsule.

Many studies have demonstrated that TGF-β signalling plays a central role in fibrotic PCO/LEC EMT, and previous work in an *in vivo* lens injury model has shown that phosphorylation of SMAD-3 is central to this process 7,39. Consistent with these reports, SMAD-3 phosphorylation is detected in wild-type LCs by 48 hrs after surgery (Fig. 9A and C) and these levels are greatly elevated by 5 days after surgery, especially in cells expressing α-SMA (Fig. 9E and G). However, αVMLR10 LCs do not exhibit elevated levels of phosphorylated SMAD-3 at either > hrs (Fig. 9B and D) or 5 days (Fig. 9F and H) after surgery.

### TGF-β–induced protein is not deposited in αVMLR10 ECM 48 hrs after surgery

**Figure 10:**
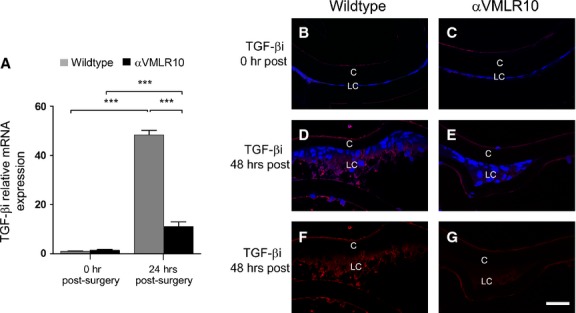
(A) RT-PCR quantification of TGF-βi mRNA levels in wild-type and αVMLR10 mice residual lens epithelial cells (LCs) on capsular bags collected at 0 and 24 hrs after surgery showing almost no expression of TGF-βi mRNA at 0 hr after surgery in both wild-type and αVMLR10 LCs, but a significant increase in mRNA levels in both wild-type and αVMLR10 LCs ***P < 0.0001. TGF-βi mRNA levels in wild-type LCs at 24 hrs after surgery were significantly higher than that of αVMLR10 LCs at 24 hrs after surgery (***P < 0.001). mRNA expression was normalized to β2M and fold change differences were calculated based on 0 hr post-surgery wild-type mRNA expression. All experiments had *N* = 5. Values are expressed as mean ± SEM. Asterisks (*) indicate statistically significant fold changes from 0 hr after surgery. (B) Immunohistochemistry results showing TGF-βi protein expression 0 hr after surgery in wild-type LCs and (C) αVMLR10 LCs. (D) TGF-βi expression in wild-type LCs at 48 hrs after surgery. (E) TGF-βi expression in αVMLR10 LCs at 48 hrs after surgery. (F) TGF-βi expression alone in wild-type LCs at 48 hrs after surgery. (G) TGF-βi expression alone in αVMLR10 LCs at 48 hrs after surgery. Scale bar 35 μm. Red = TGF-βi, blue = nucleus, LC = residual lens cells, C = lens capsule.

Transforming growth factor-β–induced protein (TGF-βi) is an ECM molecule and α_V_ integrin ligand 40, whose expression and ECM deposition robustly up-regulate in response to TGF-β signalling 41. Consistent with this, TGF-βi mRNA is robustly up-regulated in the remnant LCs of wild-type mice by 24 hrs after fibre cell removal (Fig. 10A), and obvious TGF-βi protein deposition is detectable around wild-type LCs by 48 hrs after surgery (Fig. 10D and F). In contrast, despite a significant TGF-βi mRNA up-regulation in αVMLR10 LCs, such up-regulation was significantly lower (*P* < 0.001) than that observed in wild-type (Fig. 10A); moreover, TGF-βi protein deposition was not detected in αVMLR10 LCs at 48 hrs after lens fibre cell removal (Fig. 10E and G).

## Discussion

### α_V_ integrin is not necessary for the latter stages of lens development, morphology or the maintenance of lens optical quality

Integrins are heterodimeric transmembrane proteins best known as cellular receptors for diverse ECM proteins 15. In the normal lens, the expression of α_V_ integrin and its beta integrin partners have been previously reported, while mice lacking both α3 and α6 integrin 42 or β1 integrin from the lens 16 have profound lens epithelial defects; zebrafish lacking α_5_ integrin have lens fibre cell defects 27. Here, while α_V_ integrin protein is detectable in the lens, particularly along the lateral membranes of both adult (Fig. 3) and embryonic (data not shown) lens fibres, lenses lacking α_V_ integrins are transparent and morphologically normal when examined by both light and scanning electron microscopy. However, αVMLR10 (α_V_^[−/flox]^; MLR10-*cre*) lenses were significantly larger than wild-type lenses in early adulthood, although this difference disappeared by 6 months of age. This difference does not appear to be as a result of a misregulation of water homoeostasis as has been seen in some lens pathologies 43 as the dry/wet lens ratio is unchanged. Instead, it is likely that α_V_ integrin plays a subtle role in regulating lens growth that was not revealed by proliferation analyses.

### α_V_ integrin proteins are up-regulated in residual LCs by 48 hrs after lens fibre cell removal

α_V_ integrins are a class of six distinct heterodimeric proteins α_V_β_1_, α_V_β_3_, α_V_β_5_, α_V_β_6_ and α_V_β_8_, which are known as promiscuous receptors for diverse ECM proteins associated with mesenchymal cells including fibronectin, vitronectin, tenascin-C and TGF-βi 44. α_V_ integrins commonly up-regulate during EMT/tissue fibrosis and cancer and have been proposed to play diverse roles in this process in multiple cell types 21. We found that the proteins levels of α_V_ integrin and four of five of its β-integrin partners (β1, β5, β6 and β8) are up-regulated in LCs following fibre cell removal, while β3 integrin was expressed at very low levels both before and after surgery. Although few prior investigations of α_V_ integrin expression in the lens have been published, our data are consistent with prior reports of elevated α_V_β_5_ expression in an established human lens epithelial cell line treated with TGF-β, and elevated α_V_β_6_ expression in post-operative human lens capsule and human primary LEC explants induced to undergo EMT in an *in vitro* PCO model 18,45.

However, while the protein levels of α_V_ integrins were robustly up-regulated by 48 hrs after lens fibre cell removal, the levels of α_V_ integrin mRNA and that of its β-subunits were not, suggesting that this phenomenon is regulated at the level of either protein translation or protein stability. miR-31 is a microRNA known for its ability to negatively regulate invasion–metastasis cascades in cancer progression by repressing the expression of proteins important for this process 33,46. We found abundant miR-31 in the lens epithelium consistent with a prior report 47 and a recent study demonstrated that miR-31 can repress α_V_ integrin translation by direct binding to the 3′UTR of the α_V_ integrin mRNA 34. As miR-31 levels decrease more than 50% by 24 hrs after surgery in wild-type mice (Fig. 2B), it is possible that the up-regulation of α_V_ integrin protein levels by 48 hrs after surgery is regulated *via* this down-regulation of miR-31. This postulate can be tested in the future to gain insight into the earliest events occurring in LCs following lens fibre cell removal/cataract surgery.

### αv integrin plays a crucial role in fibrotic type, but not pearl type PCO development

Clinically, two different types of PCO occur following cataract surgery, the ‘fibrotic type’ and the ‘pearl type’. The fibrotic type is typically attributed to the migration of LCs into the optical path concomitant with their EMT resulting in these cells overexpressing α-SMA, depositing mesenchymal ECM proteins and contraction of the posterior capsule leading to light scatter and visual disability 8,37,48. In ‘pearl type’ PCO, the LCs that migrate onto the posterior capsule enter the fibre cell differentiation pathway, presumably in an attempt to regenerate the lens. However, as they do not form the correct cellular organization for transparency, these cells are also light scattering 11. Finally, many designs of IOL implants used in cataract surgery seek to trap residual LECs at the lens equator, and these cells also often attempt to undergo lens fibre differentiation to form an opacity outside of the visual axis known as Soemmering's ring 49,50. Therefore, it is apparent that PCO arises from two distinct cellular responses to cataract surgery with some LCs undergoing EMT, whereas others attempt fibre cell regeneration 7,51.

As α_V_ integrins are up-regulated following fibre cell removal in a mouse model (Fig. 1), we tested the response of αVMLR10 LCs to fibre cell removal. In wild-type mice, we noticed a robust increase in residual epithelial cell proliferation along with a significant increase in α-SMA expression by 48 hrs after surgery, which is often used as a hallmark marker for LCs undergoing EMT. In addition, wild-type LCs up-regulate a number of TGF-β–associated mesenchymal ECM proteins particularly fibronectin, tenascin-C, vitronectin and TGF-βi. As all the four ECM proteins have been reported to be ligands for α_V_ integrins, these data suggest that a functional α_V_ integrin/ECM ligand network up-regulates in LCs by 48 hrs of surgery in this model. In contrast, αVMLR10 lenses, which lack α_V_ integrins do not exhibit robust LC proliferation, do not up-regulate α-SMA expression and have greatly attenuated expression of the mesenchymal ECM molecules. These data suggest that α_V_ integrins play an essential role in the early regulation of the EMT that occurs during the development of fibrotic PCO.

At later times after surgery, wild-type LCs continue to proliferate, forming additional α-SMA expressing myofibroblasts embedded in an ECM rich in fibronectin, tenascin-C and vitronectin (Fig. 7). However, by 5 days after surgery, not all cells express α-SMA and islands of cells, which instead express fibre cell markers, become obvious (Fig. 8). Notably, αVMLR10 lenses still begin expressing fibre cell markers at this time, although they still do not express appreciable levels of fibrotic markers. This suggests that α_V_ integrin does not play a role in regulating the lens regenerative pathway, which is activated after surgery 29 consistent with our observation that α_V_ integrin is also not involved in regulating normal lens fibre cell differentiation during development. Overall, these data point to a role for α_V_ integrin in regulating pathways that are critical for the establishment of fibrotic, but not pearl-type PCO.

### The loss of α_V_ integrin impairs TGF-β signalling after surgery

Treatment of LCs with TGF-β *in vitro* can induce most cellular and molecular changes associated with fibrotic PCO including myofibroblast formation, the expression of fibrotic ECM proteins, LC proliferation and capsule wrinkling 7,8,52, whereas transgenic mice overexpressing an active form of TGF-β in lens fibre cells develop anterior subcapsular cataracts, which share many features with fibrotic PCO 53.

Transforming growth factor-β can mediate its biological effects *via* canonical (SMAD2/3 dependent) signalling or through non-canonical pathways (SMAD-independent signalling). Our results show that SMAD-3 activation (SMAD-3 phosphorylation), which has been previously shown to be important for LEC EMT in a lens injury model 39,54,55, is not appreciably detected until 48 hrs after surgery in our model, coincident with the up-regulation of α_V_ integrin protein expression. Furthermore, the expression of TGF-βi, a known direct transcriptional target of pSMAD-3 40, also up-regulates at the protein level around this time frame. The levels of SMAD-3 phosphorylation then continue to increase at later times after surgery consistent with TGF-β activation increasing through 5 days after surgery in wild-type mice. Notably, no appreciable SMAD-3 phosphorylation is detected in LCs lacking α_V_ integrins (Fig. 9). Furthermore, while the mRNA levels of TGF-βi up-regulated to a certain extent in α_V_ integrin null lenses at 24 hrs after surgery, this up-regulation was insufficient to deposit detectable levels of TGF-βi protein in the ECM by 48 hrs after surgery, suggesting that α_V_ integrins are playing a fundamental role in regulating the TGF-β pathway after lens injury/cataract surgery.

### α_V_ integrins may be playing a role in activating TGF-β signalling during lens EMT

All three isoforms of TGF-β, TGF-β1, 2, and 3 are synthesized by LCs *in vivo* and the latent forms of these molecules are abundant in the aqueous and vitreous humour of the eye 56. Transforming growth factor-βs are activated by numerous cellular mechanisms, all of which result in liberation of the active TGF-β molecule from its latency-associated peptide (LAP)/Latent TGF-β–binding proteins (LTBPs). Notably, α_V_ integrins can activate latent TGF-β by at least three distinct mechanisms 57–59. α_V_ integrins can bind to an RGD sequence present in the LAP of either TGF-β1 or TGF-β3, inducing these molecules to undergo a conformational change to liberate the active TGF-β molecule 60–63. α_V_ integrins can also interact with matrix metalloproteases (MMPs), particularly MMP2 and MMP9, which tethers them to the cell surface promoting proximity of MMPs to the LAP and sequesters the large latent complex close to the type II TGF-β receptor 64–66. This interaction can bring these proteases into the proximity of latent TGF-β where they can activate TGF-β *via* proteolysis of the LAP or the LTBP. This mechanism is supported by reports that both MMP2 and MMP9 are also up-regulated in LEC EMT/PCO and have been proposed to play diverse roles in this process 67.

Alternatively, cross-talk between TGF-β and α_V_ integrin signalling can occur downstream of initial receptor activation and regulate various cellular processes 68. Signals propagated intracellularly by integrin-associated adaptor proteins such as ILK, Src, PTKs and FAK can subsequently activate other downstream TGF-β–induced EMT and cell proliferation players such as MAPK, Ras/Rho, small GTPases, PI3K and AKT 69,70. For example, α_V_ integrins can interact with the TGF-β co-receptor, endoglin, and this interaction can enhance α_V_ integrin–mediated TGF-β activation 71. Moreover, α_V_ integrins can also intracellularly associate with the type II TGF-β receptor tyrosine kinases, auto-stimulating the receptor's phosphorylation on its tyrosine, initiating a TGF-β–induced EMT 72–74. Altogether, all of these pathways may override the normal brakes on TGF-β signalling levels resulting fibrotic PCO.

## Conclusion

**Figure 11:**
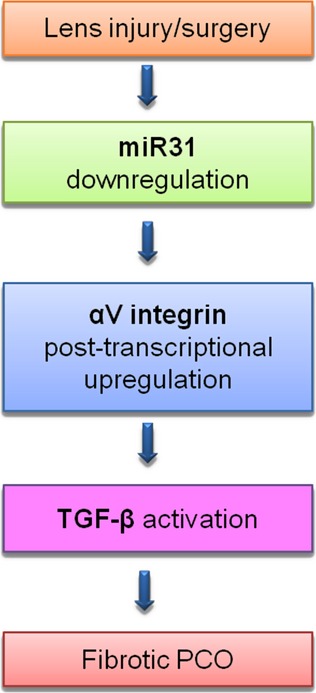
Proposed model of how α_V_ integrin could mediate TGF-β–induced epithelial–mesenchymal transition during fibrotic PCO.

In this study, we have answered three fundamental questions regarding the function of α_V_ integrins in the lens. First, we demonstrated that α_V_ integrins are not necessary for either the latter stages of lens development or the maintenance of lens morphology after lens vesicle closure. Second, we demonstrated that α_V_ integrins are required for LECs to undergo EMT following a lens injury that mimics cataract surgery, indicating that these proteins play an important role in the pathogenesis of fibrotic, but not pearl-type, PCO. Third, our data suggest that α_V_ integrins may mediate these functions by enhancing TGF-β–mediated signalling following surgery, perhaps *via* their known roles in the activation of latent TGF-β (Fig. 11).

These data suggest that fibrotic PCO may be prevented by one or more of the inhibitors of α_V_ integrin's function currently in clinical trials to treat cancer and other diseases 21.

## References

[1] Asbell PA, Dualan I, Mindel J (2005). Age-related cataract. Lancet.

[2] World Health Organization (2008). Global initiative for the elimination of blindness. WHO/PBL.

[3] Steinberg EP, Javitt JC, Sharkey PD (1993). The content and cost of cataract surgery. Arch Ophthalmol.

[4] Laroche L (2013). actuality in cataract treatment. Rev Prat.

[5] Foster A (2001). Cataract and “vision 2020-the right to sight” initiative. Br J Ophthalmol.

[6] Apple DJ, Escobar-Gomez M, Zaugg B (2011). Modern cataract surgery: unfinished business and unanswered questions. Surv Ophthalmol.

[7] de Iongh RU, Wederell E, Lovicu FJ (2005). Transforming growth factor-beta-induced epithelial-mesenchymal transition in the lens: a model for cataract formation. Cells Tissues Organs.

[8] Wormstone IM, Wang L, Liu CSC (2009). Posterior capsule opacification. Exp Eye Res.

[9] Werner L, Tassignon MJ, Zaugg BE (2010). Clinical and histopathologic evaluation of six human eyes implanted with the bag-in-the-lens. Ophthalmology.

[10] Awasthi N, Guo S, Wagner BJ (2009). Posterior capsular opacification: a problem reduced but not yet eradicated. Arch Ophthalmol.

[11] Dewey S (2006). Posterior capsule opacification. Curr Opin Ophthalmol.

[12] Hales AM, Schulz MW, Chamberlain CG (1994). TGF-β1 induces lens cells to accumulate alpha-smooth muscle actin, a marker for subcapsular cataracts. Curr Eye Res.

[13] Cousins SW, McCabe MM, Danielpour D (1991). Identification of transforming growth factor-beta as an immunosuppressive factor in aqueous humor. Invest Ophthalmol Vis Sci.

[14] Hynes RO (1992). Integrins: versatility, modulation, and signaling in cell adhesion. Cell.

[15] Hynes RO (2002). Integrins: bidirectional, allosteric signaling machines. Cell.

[16] Simirskii VN, Wang Y, Duncan MK (2007). Conditional deletion of beta1-integrin from the developing lens leads to loss of the lens epithelial phenotype. Dev Biol.

[17] Walker J, Menko AS (2009). Integrins in lens development and disease. Exp Eye Res.

[18] Sponer U, Pieh S, Soleiman A (2005). Upregulation of alphavbeta6 integrin, a potent TGF-β1 activator, and posterior capsule opacification. J Cataract Refract Surg.

[19] Zuk A, Hay ED (1994). Expression of beta 1 integrins changes during transformation of avian lens epithelium to mesenchyme in collagen gels. Dev Dyn.

[20] Barbour WK, Saika S, Miyamoto T, Ohkawa K (2004). Expression patterns of beta1-related alpha integrin subunits in murine lens during embryonic development and wound healing. Curr Eye Res.

[21] Nemeth JA, Nakada MT, Trikha M (2007). Alpha-v integrins as therapeutic targets in oncology. Cancer Invest.

[22] McCarty JH, Lacy-Hulbert A, Charest A (2005). Selective ablation of alphav integrins in the central nervous system leads to cerebral hemorrhage, seizures, axonal degeneration and premature death. Development.

[23] Zhao H, Yang Y, Rizo CM (2004). Insertion of a pax6 consensus binding site into the AlphaA-crystallin promoter acts as a lens epithelial cell enhancer in transgenic mice. Invest Ophthalmol Vis Sci.

[24] Lakso M, Pichel JG, Gorman JR (1996). Efficient *in vivo* manipulation of mouse genomic sequences at the zygote stage. Proc Natl Acad Sci USA.

[25] Shiels A, King JM, Mackay DS (2007). Refractive defects and cataracts in mice lacking lens intrinsic membrane protein-2. Invest Ophthalmol Vis Sci.

[26] Duncan MK, Cui W, Oh DJ, Tomarev SI (2002). Prox1 is differentially localized during lens development. Mech Dev.

[27] Scheiblina DA, Gaob J, Caplana JL Beta-1 integrin is important for the structural maintenance and homeostasis of differentiating fiber cells 2014.

[28] Desai VD, Wang Y, Simirskii VN (2010). Cd44 expression is developmentally regulated in the mouse lens and increases in the lens epithelium after injury. Differentiation.

[29] Call MK, Grogg MW, Del Rio-Tsonis K (2004). Lens regeneration in mice: implications in cataracts. Exp Eye Res.

[30] Reed NA, Oh D-J, Czymmek KJ (2001). An immunohistochemical method for the detection of proteins in the vertebrate lens. J Immunol Methods.

[31] Kotogany E, Dudits D, Horvath G (2010). A rapid and robust assay for detection of s-phase cell cycle progression in plant cells and tissues by using ethynyl deoxyuridine. Plant Methods.

[32] Warren M, Puskarczyk K, Chapman S (2009). Chick embryo proliferation studies using edu labeling. Dev Dyn.

[33] Valastyan S, Reinhardt F, Benaich N (2009). A pleiotropically acting microrna, mir-31, inhibits breast cancer metastasis. Cell.

[34] Augoff K, Das M, Bialkowska K (2011). Mir-31 is a broad regulator of beta1-integrin expression and function in cancer cells. Mol Cancer Res.

[35] Tanaka S, Saika S, Ohmi S (2002). Cellular fibronectin, but not collagens, disappears in the central posterior capsules during healing after lens extraction and iol implantation in rabbits. Jpn J Ophthalmol.

[36] Tanaka S, Sumioka T, Fujita N (2010). Suppression of injury-induced epithelial-mesenchymal transition in a mouse lens epithelium lacking tenascin-c. Mol Vis.

[37] Eldred JA, Dawes LJ, Wormstone IM (2011). The lens as a model for fibrotic disease. Philos Trans R Soc Lond B Biol Sci.

[38] Gwon A (2006). Lens regeneration in mammals: a review. Surv Ophthalmol.

[39] Saika S, Kono-Saika S, Ohnishi Y (2004). Smad3 signaling is required for epithelial-mesenchymal transition of lens epithelium after injury. Am J Pathol.

[40] Nam JO, Kim JE, Jeong HW (2003). Identification of the alphavbeta3 integrin-interacting motif of betaig-h3 and its anti-angiogenic effect. J Biol Chem.

[41] Jeon ES, Kim JH, Ryu H (2012). Lysophosphatidic acid activates tgfbip expression in human corneal fibroblasts through a TGF-β1-dependent pathway. Cell Signal.

[42] De Arcangelis A, Mark M, Kreidberg J (1999). Synergistic activities of alpha3 and alpha6 integrins are required during apical ectodermal ridge formation and organogenesis in the mouse. Development.

[43] Shiels A, Bassnett S, Varadaraj K (2001). Optical dysfunction of the crystalline lens in aquaporin-0-deficient mice. Physiol Genomics.

[44] Kerr JS, Slee AM, Mousa SA (2002). The alpha(v) integrin antagonists as novel anticancer agents: an update. Expert Opin Inv Drug.

[45] Dawes LJ, Elliott RM, Reddan JR (2007). Oligonucleotide microarray analysis of human lens epithelial cells: TGF-β regulated gene expression. Mol Vis.

[46] Sossey-Alaoui K, Downs-Kelly E, Das M (2011). Wave3, an actin remodeling protein, is regulated by the metastasis suppressor microrna, mir-31, during the invasion-metastasis cascade. Int J Cancer.

[47] Karali M, Peluso I, Gennarino VA (2010). Mirneye: a microrna expression atlas of the mouse eye. Bmc Genomics.

[48] van Bree MC, van der Meulen IJ, Franssen L (2011). Imaging of forward light-scatter by opacified posterior capsules isolated from pseudophakic donor eyes. Invest Ophthalmol Vis Sci.

[49] Huang YS, Xie LX (2007). Lens fiber differentiation in rats posterior capsule opacification. Zhonghua Yan Ke Za Zhi.

[50] Kappelhof J, Vrensen G, Jong P (1987). The ring of soemmerring in man: an ultrastructural study. Graefes Arch Clin Exp Ophthalmol.

[51] Marcantonio JM, Vrensen GF (1999). Cell biology of posterior capsular opacification. Eye (Lond).

[52] Dawes LJ, Sleeman MA, Anderson IK (2009). TGF-β/smad4-dependent and -independent regulation of human lens epithelial cells. Invest Ophthalmol Vis Sci.

[53] Lovicu FJ, Schulz MW, Hales AM (2002). TGF-β induces morphological and molecular changes similar to human anterior subcapsular cataract. Br J Ophthalmol.

[54] Saika S, Okada Y, Miyamoto T (2001). Smad translocation and growth suppression in lens epithelial cells by endogenous TGF-β2 during wound repair. Exp Eye Res.

[55] Saika S, Miyamoto T, Ishida I (2002). TGF-β-smad signalling in postoperative human lens epithelial cells. Br J Ophthalmol.

[56] Lee EH, Joo C-K (1999). Role of transforming growth factor-β in transdifferentiation and fibrosis of lens epithelial cells. Invest Ophthalmol Vis Sci.

[57] Wipff P-J, Rifkin DB, Meister J-J (2007). Myofibroblast contraction activates latent TGF-β1 from the extracellular matrix. J Cell Biol.

[58] Wipff P-J, Hinz B (2008). Integrins and the activation of latent transforming growth factor [beta]1 - an intimate relationship. Eur J Cell Biol.

[59] Mamuya FA, Duncan MK (2012). α_V_ integrins and TGF-β–induced EMT: a circle of regulation. J Cell Mol Med.

[60] Munger JS, Huang X, Kawakatsu H (1999). A mechanism for regulating pulmonary inflammation and fibrosis: the integrin [alpha]v[beta]6 binds and activates latent TGF-β1. Cell.

[61] Annes JP, Rifkin DB, Munger JS (2002). The integrin [alpha]v[beta]6 binds and activates latent tgf[beta]3. FEBS Lett.

[62] Annes JP, Chen Y, Munger JS (2004). Integrin [alpha]v[beta]6-mediated activation of latent TGF-β requires the latent TGF-β binding protein-1. J Cell Biol.

[63] Ludbrook SB, Barry ST, Delves CJ (2003). The integrin alphavbeta3 is a receptor for the latency-associated peptides of transforming growth factors beta1 and beta3. Biochem J.

[64] Brooks PC, Stromblad S, Sanders LC (1996). Localization of matrix metalloproteinase mmp-2 to the surface of invasive cells by interaction with integrin alpha v beta 3. Cell.

[65] Rolli M, Fransvea E, Pilch J (2003). Activated integrin αvβ3 cooperates with metalloproteinase mmp-9 in regulating migration of metastatic breast cancer cells. Proc Natl Acad Sci USA.

[66] Mu D, Cambier S, Fjellbirkeland L (2002). The integrin αvβ8 mediates epithelial homeostasis through mt1-mmp–dependent activation of TGF-β1. J Cell Biol.

[67] West-Mays JA, Pino G (2007). Matrix metalloproteinases as mediators of primary and secondary cataracts. Expert Rev Ophthalmol.

[68] Cary LA, Han DC, Guan JL (1999). Integrin-mediated signal transduction pathways. Histol Histopathol.

[69] Dedhar S (1999). Integrins and signal transduction. Curr Opin Hematol.

[70] Zhang YE (2009). Non-smad pathways in tgf-beta signaling. Cell Res.

[71] Rivera LB, Brekken RA (2011). Sparc promotes pericyte recruitment *via* inhibition of endoglin-dependent TGF-β1 activity. J Cell Biol.

[72] Guo W, Giancotti FG (2004). Integrin signalling during tumour progression. Nat Rev Mol Cell Biol.

[73] Galliher A, Schiemann W (2006). Beta3 integrin and src facilitate transforming growth factor-beta mediated induction of epithelial-mesenchymal transition in mammary epithelial cells. Breast Cancer Res.

[74] Playford MP, Schaller MD (2004). The interplay between src and integrins in normal and tumor biology. Oncogene.

